# Sexual Dimorphism in Crowned Lemur Scent-Marking

**DOI:** 10.3390/ani11072091

**Published:** 2021-07-14

**Authors:** Emily J. Elwell, David Walker, Stefano Vaglio

**Affiliations:** 1 Department of Biology, Chemistry and Forensic Science, University of Wolverhampton, Wolverhampton WV1 1LY, UK; E.J.Elwell@wlv.ac.uk (E.J.E.); D.Walker7@wlv.ac.uk (D.W.); 2 Department of Anthropology & Behaviour, Ecology and Evolution Research (BEER) Centre, Durham University, Durham DH1 3LE, UK

**Keywords:** communication, olfaction, scent-marking, gas chromatography–mass spectrometry, *Eulemur coronatus*

## Abstract

**Simple Summary:**

Primates are typically thought to use hearing and vision more than the sense of smell. However, lemurs show a complex olfactory repertoire which includes conspicuous scent-marking behaviours (i.e., a form of olfactory communication displayed by animals that deposit their odour in specific places to transmit a message to other animals). We studied two pairs of crowned lemurs at Colchester and Twycross zoos (UK) by combining behavioural observations and chemical analyses of odour secretions released via scent-marking. Male lemurs scent-marked most frequently, showing three types of behaviours: ano-genital marking for applying their scent onto females; head marking for placing their secretions on or near the mark left by another individual; and wrist marking to deposit their mark in specific meaningful areas of the enclosure. Female lemurs displayed only ano-genital marking, primarily on feeding devices. We detected a total of 38 volatile compounds in male ano-genital scent-marks and 26 in female ano-genital odour secretions, including many compounds that have been identified in odour profiles of other primates. In conclusion, we found sexual dimorphism in crowned lemur scent-marking. In males, head and wrist marking behaviours would play defensive territorial functions, while ano-genital marking may be related to socio-sexual communication; female ano-genital marking could be involved in resource defense. This study contributes to improving our understanding of lemur communication.

**Abstract:**

Primates are traditionally considered to have a poor sense of smell. However, olfaction is important for non-human primates as demonstrated by conspicuous scent-marking behaviours in lemurs. We studied two pairs (*n =* 4) of crowned lemurs (*Eulemur coronatus*) housed at Colchester and Twycross zoos (UK) by combining behavioural observations and chemical analyses of scent-marks and glandular swabs. We recorded observations of olfactory behaviours for 201 h using instantaneous scan sampling. We investigated the volatile compounds of ano-genital odour secretions (*n =* 16) using solid-phase microextraction and gas chromatography-mass spectrometry. Males scent-marked most frequently, displaying ano-genital marking for allomarking, head marking for countermarking and wrist marking in specific areas of the enclosure. Females displayed ano-genital marking, predominantly on feeding devices. We detected a total of 38 volatile components in all male ano-genital scent-marks and 26 in all female samples of ano-genital odour secretions, including a series of esters, aldehydes, ketones, alcohols, terpenes, volatile fatty acids and hydrocarbons that have been identified in odour profiles of other primates. In conclusion, we found sexual dimorphism in crowned lemur scent-marking. Male head and wrist marking behaviours might play defensive territorial functions, while ano-genital marking would be related to socio-sexual communication as chemical mate-guarding. Female ano-genital marking might be involved in resource defense.

## 1. Introduction

Communication plays a vital role for many species and is particularly important in those that exhibit complex social systems [1]. Animals display different types of behaviours to relay messages to other individuals, including visual [2], auditory [3] and olfactory [4] modes of communication. The olfactory system is, from an evolutionary perspective, the oldest sensory system and is found in many different species including mammals, reptiles and birds [5]. Olfactory communication has several benefits over other communication forms, as chemical signals can be received in the absence of the sender and are then able to persist in the environment [4,6]. Chemical signals are also thought to be honest indicators of health conditions as they are directly linked to internal hormones and energetically costly to produce [6].

In mammals, including primates, olfactory communication plays an important role in both solitary and social species [5,7]. Olfaction has functions in foraging [8], territorial defense [9] and reproduction [10]. In particular, scent signals can provide information on individual identity, ranks, social status, age, sex and reproductive status [6,11,12] in several mammal species. Scent signals can come from specialised glands, skin, sweat, urine and faeces [13], and these signals can be mixed to provide specific messages [14]. Olfactory signals are complex and can be made up of various types of compounds, including both volatile compounds, and non-volatile ones such as lipocalin proteins [15]. Scent-marking is a common eye-catching olfactory behaviour observed in many different terrestrial species, including mammals, such as rodents and felines, and reptiles [16]. Mammals tend to leave scent-marks, urine or feaces, in meaningful locations, often along boundaries of their territories [17].

Primates have typically been considered to be microsomatic and to instead rely mainly on visual and auditory senses [18,19]. However, there is increasing evidence to suggest that olfaction plays a more important role in many primate species than previously thought [11,20,21,22]. Similar to other mammalian species, olfaction has reproductive, social and identification functions in primates. Both strepsirrhines and platyrrhines show extensive use of olfactory and scent-marking behaviours, while this has been less commonly reported in catarrhines.

Several species of lemur possess scent-glands; this is the case with ring-tailed lemurs (*Lemur catta*), *Eulemur* species [13], Milne–Edwards’s sifaka (*Propithecus edwardsi*) and red-ruffed lemurs (*Varecia variegata rubra*) [12]. Furthermore, some species, such as ring-tailed lemurs, have more than one scent-gland type and these can be sexually dimorphic [13]. Scent-marking behaviour (i.e., a range of conspicuous behaviours displayed by individual mammals, and other terrestrial vertebrates as well, which deposit glandular secretions, otherwise faeces or urine, at meaningful places in their territories [11]) are observed extensively in lemur species, with ring-tailed lemurs often being used as a model species for olfactory communication [6]. Lemur scent-marking has many functions including marking territory, social communication and advertising reproductive status [6]. The frequency and types of scent-marking have been shown to differ between sexes [12,23] and when comparing breeding and non-breeding seasons [7,24,25] in several lemur species. Chemical analysis of lemur scent-marks has identified more than 200 volatile compounds, including over 120 compounds found in ring-tailed lemur scents alone [13,26], and shown that the volatile chemical profile of lemur scents may convey information about the sex of the signaller [12,26].

The crowned lemur (*Eulemur coronatus*) is a small, sexually dimorphic species of lemur found in the northern forests of Madagascar and the only lemur to inhabit the Cap d’Ambre Peninsula [27]. It is the smallest species in the *Eulemur* genus [13] and is currently classified as endangered by the IUCN Red List, with wild populations continuously declining [27]. Crowned lemurs show a polygynous mating system and live in multi-male multi-female groups of up to 15 individuals, but typically groups are made up of five or six individuals [28,29]. The species is one of only three *Eulemur* species which exhibit female dominance [29]. Crowned lemurs communicate mainly via vocalisations, but they also have different scent-glands; it is therefore thought that olfactory communication has a crucial role in their communication [13,24,30].

The overarching aim of this preliminary study was to improve our understanding of the olfactory behavioural repertoire, particularly focusing on the different types of scent-marking behaviours, in captive crowned lemurs. On the basis of prior work on olfactory communication in captive crowned lemurs [24] and evidence that both scent-marking and the volatile chemical profile of scents are sexually dimorphic in well-studied lemur species [19,26], we predicted that scent-marking behaviours and the chemical composition of scent-marks would differ between sexes. We examined variation in scent-marking behaviours and related this to the location of deposition of scent-marks and to the sex of the signaller. We also described the chemical composition of male ano-genital scent-marks and female ano-genital glandular swabs, and highlighted promising areas of future research work.

## 2. Materials and Methods

### 2.1. Subjects and Housing

We studied two captive groups of crowned lemurs (*n =* 4) housed at Twycross and Colchester zoos. The pair at Twycross Zoo consisted of an adult female (aged 8 years at the beginning of the study period) and an adult male (aged 6 years). The pair at Colchester also consisted of an adult female (aged 6 years) and an adult male (aged 6 years). Crowned lemurs are considered sexually mature at 20 months old [31]. The female at Twycross Zoo was contracepted. Both pairs were in non-breeding season during the study period.

We carried out behavioural observations and odour sampling from July to November 2019 at Twycross and Colchester zoos. At both institutions the troops lived in heated indoor enclosures with access to outdoor enclosures. The outdoor enclosure at Colchester Zoo was a visitor walkthrough enclosure where crowned lemurs were co-hosted with other lemur species including ring-tailed and red-bellied lemurs.

### 2.2. Behavioural Data Collection and Analysis

We collected behavioural data by using instantaneous scan sampling [32]. Behaviours were recorded every 30 s in one-hour intervals three times per day (once in the morning, once midday, once in the afternoon) over a period of four months. We also used ad libitum sampling for recording of olfactory behaviours (Table 1), including scent-marking type (i.e., ano-genital marking, head marking, wrist marking) and the location of deposition (i.e., conspecific, countermark, branches, climbing frames, enrichment objects, hatches, ground) (Table 2). We recorded a total of 201 h of observations throughout the study period (132 h at Twycross Zoo; 69 h at Colchester Zoo) which included 360 scan samples per individual each day.

We investigated the relationship between sex and olfactory behaviours. We also investigated the differences in function and deposition location of scent-marks. We performed both Mann–Whitney U tests and Kruskal–Wallis tests followed by post-hoc pairwise Mann–Whitney U tests. A significance level of *p* < 0.05 was applied. All statistical analyses were carried out in SPSS software version 26 (IBM, London, UK) [33].

### 2.3. Odour Sampling and Analysis

We collected male odour secretions as soon as they were released via spontaneous scent-marking (12 samples in total) on brand-new sterile filter paper (circles, diameter 70 mm; Whatman^®^, Little Chalfont, UK) that was fixed via paper tape to hatches, branches and climbing structures in both the indoor and outdoor enclosures at Twycross (7 samples) and Colchester (5 samples) zoos. Odour secretions were also collected from the female at Twycross Zoo during a veterinary check, with sterile filter paper rubbed across the ano-genital area 10 times using steady pressure (4 samples). We were not able to collect any odour samples from the female at Colchester Zoo. We also exposed control filter paper to the air of the enclosures and veterinary environments in order to identify any volatile compounds that did not derive from crowned lemurs. All samples were placed into sterile vials, sealed and immediately stored at −20 °C. We used 10 mL screw-capped clear glass vials (thread 180.D. 22.5 X H 46 mm) closed by teflon-faced rubber septa and seals (1.3 mm thick).

We conducted laboratory analyses at the Rosalind Franklin Science Centre, University of Wolverhampton, UK. We investigated the volatile compounds found in the odour secretions of crowned lemurs by using solid-phase microextraction and gas-chromatography-mass-spectrometry, applying the same methods used in previous work on lemur and mandrill odour signals (reviewed in [34]).

We introduced a 65 μm polydimethylsiloxane/divinylbenzene solid-phase microextraction syringe needle through the vial septum and exposed the fibre to the headspace above the sample in the vial for 15 min at 40 °C. we analysed the adsorbed volatile analytes of all samples by using a 5975C mass spectrometer (Agilent Technologies, Santa Clara, CA, USA) EI, 70 eV, coupled directly to a 7890B gas chromatograph (Agilent Technologies) equipped with a fused silica HP5-MS UI capillary column (Agilent Technologies) 30 m × 0.25 mm crossbonded 5%-phenyl-95%-dimethylpolysiloxane, film thickness 0.25 μm. We maintained the injector temperature at 270 °C and the transfer line temperature at 280 °C. We made injections in spitless mode (purge valve opened after 1 min) with a constant flow of helium carrier gas of 1 mL per minute. We started the oven temperature program at 45 °C for 2 min, then raised it by 4 °C per minute to 170 °C and then by 20 °C per minute to 300 °C.

We assessed any potential contamination from the lab environment through blank analyses of an empty 10 mL vial (Supelco, Bellefonte, PA, USA), as well as from the enclosure and veterinary sampling sites, following the same procedure used for the lemur samples. We also conditioned the fibre at 260 °C pre-and post-injection for 5 and 20 min respectively to avoid any possible carry-over effects.

We tentatively identified eluted compounds by comparing the experimental spectra with the spectra provided by the mass-spectral library in ChemStation (Agilent Technologies) and NIST (National Institute of Standards and Technology) Database, version MSDF.01.01.2317 (Agilent Technologies). We accepted a probable identification when the minimum matching factor was higher than 80%. If more than one compound was a good match for the same GC peak we considered the chromatographic retention time and compared it with those reported in the literature for the same chromatographic column type to minimize the chances of misidentification. We analysed all samples in a short period of time to minimize interassay variability. Compounds that appeared in environmental (enclosure or veterinary) controls or the lab blanks were considered to be contaminants and removed from the results.

## 3. Results

### 3.1. Behavioural Observations

**Figure 1:**
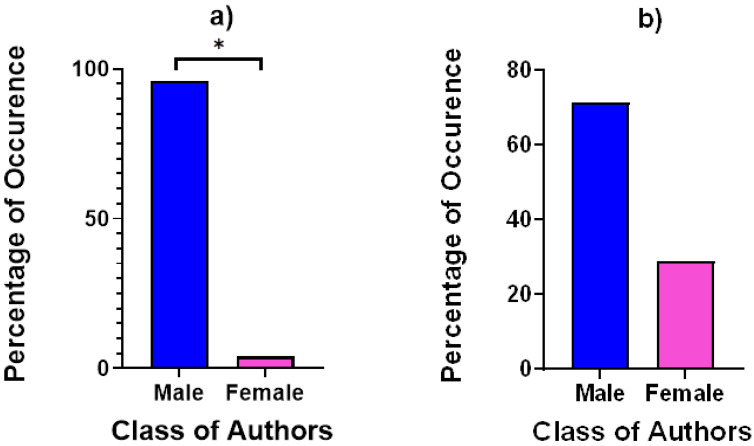
(**a**) Percentage of occurrence for total scent-marks associated to classes of authors. The difference between the sexes was significant. (**b**) Percentage of occurrence for investigative olfactory behaviours associated to classes of authors. A * indicates where significant differences between the sexes were found.

During the study period, olfactory behaviours were exhibited significantly more (U = 135.5; *p* < 0.001) by male (83.12%) than female (16.88%) individuals. This included scent marking behaviours and olfactory investigations (sniffing/licking of the environment or a conspecific and self-licking). In particular, scent-marking was significantly most commonly exhibited (U = 5218; *p* < 0.001) by males (93.04%) compared to females (6.96%) (Figure 1a). A total of 1086 scent-marks were recorded throughout the study period. Investigative behaviours were also more commonly displayed by males (71.26%) compared to females (28.74%), but this difference was not significant (Figure 1b).

The fact that both pairs were studied during the non-breeding period should have avoided any potential effects on the rate of male sniffing and allomarking across the two zoos due to the contraception of one female, but not the other one.

Females exhibited only one type of scent-marking (ano-genital marking), while males displayed three types of scent-marking behaviours (ano-genital, head and wrist marking). Males predominantly displayed ano-genital marking, followed by head marking and wrist marking (ano-genital: 66.18%; head: 21.61%; wrist: 12.21%; χ^2^ = 30.10; *p* < 0.001).

**Figure 2:**
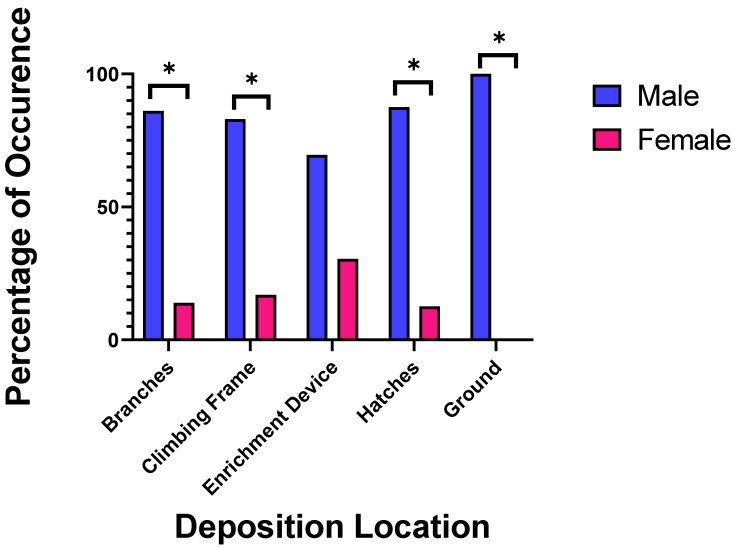
The percentage of total ano-genital scent-marks deposited on each location associated with sex. A * indicates where significant differences between the sexes were found.

The study subjects deposited scent-marks more often in the indoor than in the outdoor enclosures but this difference was not significant ((62.78%); U = 328.5; *p* = 0.13). Within enclosures scent-marks were most commonly deposited on trees and branches (45.77%) followed by enrichment devices (17.20%). We found a significant difference in ano-genital marking on branches (U = 7615; *p* = 0.002), hatches (U = 7623, *p* < 0.001), the ground (U = 8375, *p* = 0.002) and climbing structures (U = 8284, *p* = 0.033) between the sexes (Figure 2). Scent-marks were also deposited on enrichment devices, but we found no significant differences between the sexes (U = 8641, *p* = 0.122).

Only males displayed both allomarking and countermarking behaviours. Females predominantly marked enrichment devices, followed by branches/trees (devices: 40.32%; branches/trees: 35.48%; χ^2^ = 14.76; *p* = 0.002). Females also deposited scent-marks on climbing structures and hatches, but no allomarking or countermarking behaviours were observed.

**Figure 3:**
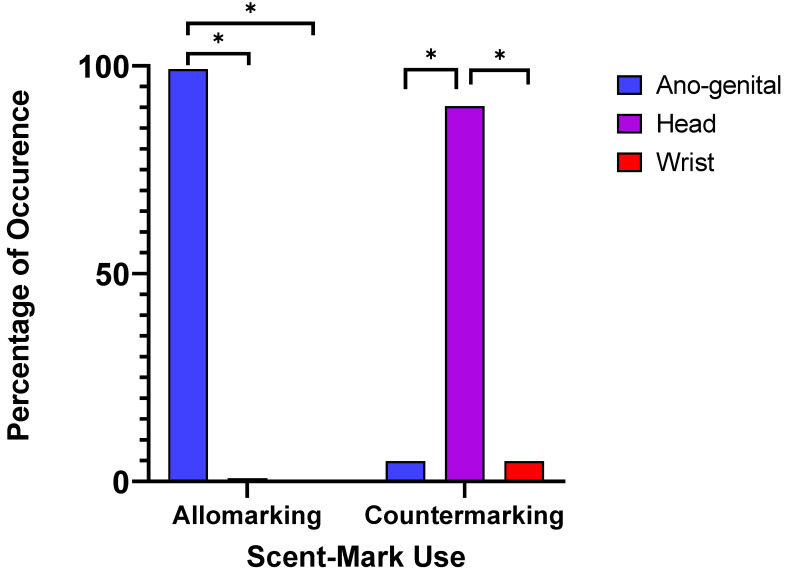
Percentages of occurrence for male allomarking and countermarking associated with scent-mark type. A * indicates where significant differences between the scent-mark types were found.

We found a preference in scent-mark type in relation to both allomarking and countermarking responses by males (Figure 3). Allomarking was most commonly observed in relation to ano-genital marking (98.58%) compared to head marking (1.42%) and wrist marking (0%), whereas countermarking was most common via head marking (90.32%) compared to ano-genital marking (4.84%) and wrist marking (4.84%).

**Figure 4:**
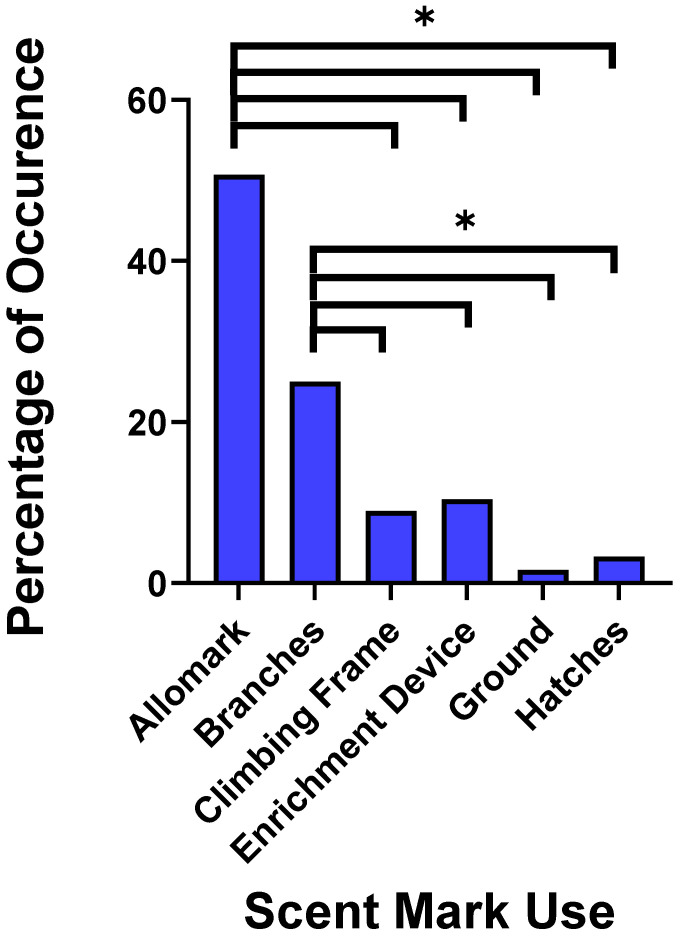
Percentages of occurrence for the use of male ano-genital marking. Allomarking accounted for the majority of ano-genital marking, followed by deposition on branches. A * indicates where significant differences between the scent-mark uses were found.

We found a significant difference (χ^2^ = 65.54; *p* < 0.001) in the use of male ano-genital scent-marks (Figure 4). Allomarking was the most frequent (50.73%) followed by deposition on branches/trees (25.00%) and enrichment devices (10.40%). Males also deposited ano-genital marks on climbing structures, hatches and the ground.

We found that the use of head marking by males was predominantly used for countermarking, followed by marking the ground and branches (countermarking: 38.36%; ground: 24.66%; branches: 13.01%; χ^2^ = 39.94; *p* < 0.001). Males also deposited head marks on climbing structures, hatches, enrichment devices and also used head marks as a form of allomarking. We found that wrist marks were mainly deposited on branches, followed by climbing structures and hatches (branches: 51.47%; structures: 23.53%; hatches: 20.59%; χ^2^ = 15.42; *p* = 0.001). In addition to this, wrist marking was occasionally used as a countermarking response.

### 3.2. Odour Secretions

**Figure 5:**
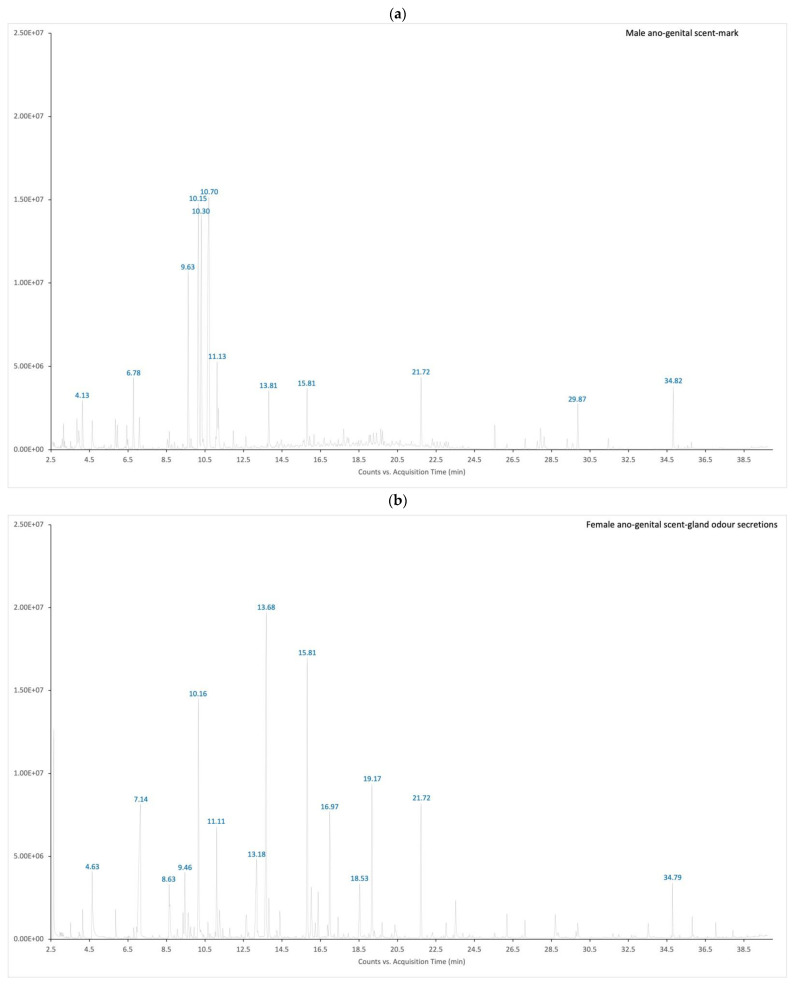
Example chromatogram from (**a**) male crowned lemur, ano-genital scent-mark; and (**b**) female crowned lemur, ano-genital scent-gland odour secretions.

We found a total of 38 distinct peaks in 12 paper samples of male ano-genital scent-marks and 26 distinct peaks in 4 paper samples of female ano-genital odour secretions; these individual peaks were present in all male (38 peaks) and female (26 peaks) paper samples but not in the controls. In total we found 56 volatile components, as 8 compounds (hexanal, nonanal, decanal, 2,2,4-trimethyl-1,3-pentanediol diisobutyrate, benzaldehyde, 1-octen-3-ol, D-limonene, 2-ethyl- 1-hexanol) were found in both male and female paper samples (Table 3). We were able to tentatively identify 42 compounds, while 14 compounds were classified as “unknown hydrocarbons” (Table 3). These compounds included a series of organic aliphatic acid esters, acetate esters, aldehydes, ketones, alcohols, terpenes, volatile fatty acids and hydrocarbons. Figure 5 shows typical chromatograms used to compare control and lemur odour samples.

## 4. Discussion

Primates have traditionally been considered “microsmatic” [35,36] (i.e., having a reduced olfactory sense [37]), with more reliance on other sensory modalities such as vision [38,39,40]; however, several studies suggest that odour may play a crucial role in both strepsirrhine and catarrhine species, e.g., [38,39,40,41,42,43,44,45,46,47,48,49]. In particular, strepsirrhines are known to rely on olfactory communication in numerous contexts, such as foraging, territorial defense, kin and group member recognition, as well as reproductive functions [10,50,51,52,53]. Importantly, studies have recently accumulated on chemical communication in non-human primates; therefore, semiochemical data are now accessible for several species, including various strepsirrhines (galagos [54]; lemurs [55,56,57], owl monkeys [58], marmosets and tamarins [59,60,61]) but also catarrhines (macaques [62], mandrills [11,63,64] and baboons [65]). In this preliminary study we focused on scent-marking behaviour, by combining behavioural observations with odour sampling, in two pairs of zoo-housed crowned lemurs.

We found that males displayed significantly more olfactory behaviours than females, including both investigative and scent-marking behaviours. Males sniffed females’ genitals significantly more than vice versa and males often allomarked females after sniffing them. Rates of sniffing of conspecifics and of genital sniffing were also found to be higher in male ring-tailed lemurs compared to females [64]. Furthermore, we found that males scent-marked significantly more than females, which supports our prediction that scent-marking in individuals varies by sex. Similarly, this has been seen in other lemur species, such as Milne–Edwards’s sifakas [66], ring-tailed lemurs [25,66], red-bellied lemurs (*Eulemur rubriventer*) [23] and red-ruffed lemurs [12], but also in non-lemur strepsirrhines, such as tamarins [60], and catarrhines, such as mandrills [11,63]. Despite the fact that one of the females was contracepted could have had an effect on the rate of male sniffing and allomarking across the two zoos, since both pairs were studied during the non-breeding period this should not have affected the behavioural data. In addition, despite Colchester Zoo included a shared outdoor enclosure, but not Twycross Zoo, we did not find any significant variation in scent-marking rates across the two sites.

Females only displayed ano-genital marking while males displayed three different types of scent-gland marking (ano-genital, head and wrist marking), as already reported by other authors [24]. This shows sexual dimorphism in scent-marking in crowned lemurs. However, despite the presence of three types of scent-glands, ano-genital marking was the most commonly observed form of scent-marking and was displayed by both sexes. This is similar to what has been found in other primate species, including the mongoose lemur (*Eulemur mongoz*) [67] and both moustached (*Saguinus mystax*) and saddle-back tamarins (*Saguinus fuscicollis*) [68].

In addition to dissimilar types of scent-marking behaviours, both the differences in scent-marking frequency and deposition location between the sexes indicate sexual dimorphism in crowned lemur scent-marking. Females marked enrichment devices more frequently than males, and interestingly only marked feeding devices (i.e., a puzzle feeder and a log with holes filled by fruits or insects) rather than other enrichment types (i.e., logs, large branches, small wooden planks and climbing structures made by ropes). This could suggest a role of scent-marking in female resource defense, which has been proposed to occur in both female Verreaux’s sifakas (*Propithecus verreauxi*) [69] and ring-tailed lemurs [70]. Female sifakas were shown to have a preference for scent-marking food trees compared to males, as well as mainly marking in areas where their home range overlapped with other groups [69]. Similarly, female ring-tailed lemurs marked significantly more frequently in zones of confrontation with other troops, which was where they also did the majority of their feeding [70]. Furthermore, the remarkable differences between the depositions of scent-marks from different male glands indicate that odour secretions may provide different signals and thus male scent-marking behaviours may have different functions. In particular, male ano-genital marking was deposited most frequently on conspecifics (allomarking), while countermarking and deposition on branches were most frequently observed for head marking and wrist marking, respectively.

Allomarking from males via ano-genital glands was the most frequent scent-marking behaviour observed throughout the study period. Importantly, this behaviour was only ever directed from males to females. Allomarking has been suggested to be a form of chemical mate guarding in wild saddle-back tamarins where, during consortship, only the mate-guarding male was observed to allomark the female, despite all males marking the same female during other periods [71]. Owl monkeys (*Aotus nancymaae*) have also been observed to allomark their conspecifics in the wild, but both males and females exhibit the behaviour [72]. Mongoose lemurs are another Eulemur species which also exhibits allomarking, directed only from male to female, similarly to our findings [67]. Wild male mongoose lemurs were observed to allomark females with their head and cheeks after they had deposited scent-marks in the environment and it has been suggested that this is a way for the monogamous species to reinforce their social bonds [67]. Captive male black lemurs (*Eulemur macaco*) have been observed to allomark females more frequently than depositing scent-marks in the environment, with certain females receiving allomarks more frequently than others [73]. However, captive male brown lemurs (*Eulemur fulvus*) scent-marked the environment more often and the majority of allomarking was performed by a dominant male [73]. Sometimes male allomarking also caused agonistic behaviour directed from the female to the male, which was also observed by other authors [24].

We found a total of 38 volatile compounds present in all male samples of odour secretions released via ano-genital scent-marking, and 26 volatile compounds present in all female samples of ano-genital scent-gland odour secretions. These low amounts of compounds in comparison to ano-genital marks by other lemur species (e.g., ring-tailed lemurs and sifakas; [26,59]) could be due to the female non-breeding season [6] and contraception [74], as these factors can alter significantly the lemur genital odorants, and to the captive setting, including lack of extra-group conspecific competitors and less variation in terms of diet and resource availability. Additionally, some of the differences observed between volatile chemical profiles across the sexes may be explained by the different types of odour samples examined (reviewed in [75]); i.e., male scent-marks deposited naturally on filter paper and female glandular swabs collected by zoo veterinarians.

The volatile chemical profile of male ano-genital odour secretions encompassed several hydrocarbons. Volatile hydrocarbons have previously been identified in odorants deriving from strepsirrhines such as ring-tailed lemurs [2], red-ruffed lemurs [12] and Coquerel’s sifakas (*Propithecus verreauxi coquereli*) [56], and catarrhines such as mandrills (*Mandrillus sphinx*) [11,63] and olive baboons (*Papio anubis*) [65]. Notably, hydrocarbon-based semiochemicals could be involved in chemical communication of genetic relatedness and thus play a role in kin recognition and inbreeding avoidance in mammals, including other lemur species [19]. In addition, we found several unknown hydrocarbons which were high-molecular-weight, less volatile hydrocarbons that might act as a fixative which slows the release of more volatile compounds, as seen in cotton-top tamarins (*Saguinus oedipus*) [76]. Male scent-marks also included methyl ketones, which are found as putative semiochemicals in aye-ayes [77] and red-ruffed lemurs [12], and volatile fatty acids, which contribute to baboon vaginal odour [65] and human body odour originating from the apocrine sweat glands [78].

On the other hand, the volatile chemical profile of female ano-genital odour secretions comprised a lower amount, but a more diverse range, of components. In particular, we identified the compound phenol which has been found in vaginal odour secretions of several primate species (e.g., ayes-ayes [77], red-ruffed lemurs [12], hamadryas baboons (*Papio hamadryas*) [79] and olive baboons [65]) and also serves as the locust phase change pheromone produced by gut bacteria [80]. Furthermore, ethyl phenol occurs in mammal urine as part of a multicomponent signal of mate attraction or range occupation (reviewed in [81]) and in ano-genital scent-marks by red-ruffed lemurs [12]. Finally, other compounds, such as α-pinene and α-terpineol, have also been identified in ruffed lemur ano-genital scent-marks [12] but are known to derive from plants and flowers; they could therefore be a by-product and vary with the environmental context.

A total of 8 compounds were identified in both male and female odour samples. Among them, benzaldehyde could play a role in signalling individual quality in crowned lemurs. This compound is considered a crucial putative semiochemical occurring at all ancestral nodes leading to both urine and glandular markers in many strepsirrhine species [82]. In particular, it has been found in scent-gland secretions released by several primates (i.e., ayes-ayes (*Daubentonia madagascariensis*) [77], red-ruffed lemurs [12], common marmosets [83], capuchin monkeys [84], emperor tamarins [60,85], Weddell’s saddleback tamarins [60,85], owl monkeys [72], mandrills [11,63] and olive baboons [65]) and it has also been reported acting as a cue to genetic quality and as a pheromone in non-primate mammals and other vertebrates (reviewed in [86]). Moreover, hexanal and 1-octen-3-ol are encountered in scent-marks in several non-primate mammals (e.g., lions (*Panthera leo*), African wild dogs (*Lycaon pictus*), gray wolves (*Canis lupus*), house mice (*Mus musculus*), red foxes (*Vulpes vulpes*)) (reviewed in [86]) and in ano-genital odour secretions in primates (e.g., aye-ayes [77], red-ruffed lemurs [12], ring-tailed lemurs [26], Coquerel’s sifakas [56], common marmosets [83], olive baboons [65]). Lastly, decanal is a key molecule of the citrus scent of seabirds (e.g., crested auklet (*Aethia cristatella*) and its concentration seems to correlate with male social rank; 2-ethyl-1-hexanol appears to be associated with fragrancies and has been found in ruffed lemur ano-genital scent-marks [12]; while D-limonene derives from leaves and flowers and is used by male insects to display successfully and attract females [87].

Overall, our chemical investigation of ano-genital odour secretions supports the hypothesis of sexual dimorphism in crowned lemur scent-marking and, more specifically, suggests that ano-genital marking might convey information about the sex of crowned lemurs. Short-range odour signals may be important in species and sex recognition; particularly, female mammals may choose male partners using chemosensory cues to their quality, which reflects factors such as their social status, richness of diet, reproductive state, health and further environmental conditions (reviewed in [86]). Male crowned lemurs might then use ano-genital allomarking to mate guard adult reproductive females and thus advertise themselves as competitors to other males (male-male indirect competition; i.e., intra-sexual selection) or even as potential mates to females (female choice; i.e., inter-sexual selection).

Finally, we have to acknowledge some major limitations of our work. First of all, we focused on an extremely restricted sample size (two adult males and two adult females) and examined a very limited number of odour samples (12 male and four female samples). Furthermore, we have to recognize the potential confounding effects related to data collected under different conditions across sites (i.e., Colchester Zoo included a shared outdoor enclosure, but not Twycross Zoo; the study subjects at Twycross Zoo were observed for almost twice the amount of time as the subjects at Colchester Zoo) and to odour sampling conducted via different types of chemical samples collected across sexes (i.e., male ano-genital scent-marks deposited naturally on filter paper and female ano-genital glandular swabs collected during a veterinary check). Overall, therefore, our findings should be viewed as pilot data.

## 5. Conclusions

In conclusion, this study supports the hypotheses that crowned lemur scent-marking differs between the sexes and may serve different functions. In particular, our findings suggest that scent-marking could play a role in intergroup spacing and intrasexual competition in this lemur species. In particular, male head and wrist marking would serve a defensive territorial function, with head marking specialized for countermarking, while male ano-genital marking would play a role in socio-sexual communication as chemical mate-guarding of female conspecifics or in reinforcing social bonds between males and females; conversely, female ano-genital marking could be involved in resource defense. Furthermore, our semiochemical investigation suggests that ano-genital odour secretions might convey information about the sex of individuals.

This study improves our understanding of the role played by scent-marking in lemur communication. However, as we focused on only four animals living in two captive pairs and due to the discrepancy of the data collected, this study must be considered a preliminary work for crowned lemurs. Future research should focus on a larger sample size and involve sampling of odour secretions released by male head and wrist marking. In addition, further studies could carry out more detailed analyses of the ratios of individual volatile components, as well as examine non-volatile components such as high-molecular-weight compounds which may extend the persistence of volatile signals in scent-marks [88,89]. Finally, it would be crucial to study the perception by recipients; for instance, looking for evidence of behavioral or physiological responses facilitated by scent-marks via bioassay tests [90].

## Figures and Tables

**Table 1:** Ethogram (based on [9,21], modified).

Behaviour	Description
Scent-Marking: Ano-genital	Individual rubs its genital region against a conspecific, the substrate or object in the enclosure.
Scent-Marking: Head	Individual rubs its head against a conspecific, the substrate or object in the enclosure.
Scent-Marking: Wrist	Individual rubs its wrists or hands against a conspecific, the substrate or object in the enclosure.
Scent-Marking: Allomark	A scent-mark placed directly on the body of another individual using either ano-genital, head or wrist glands.
Scent-Marking: Countermark	A scent-mark placed onto the location another individual scent-marked within 30 s using either ano-genital, head or wrist glands.
Sniffing/Licking: Environment	Individual deliberately places its nostrils/tongue within 3 cm from the substrate or object and sniffs/licks
Sniffing/Licking: Conspecific	Individual deliberately places its nostrils/tongue within 3 cm from a conspecific and sniffs/licks.
Self-Licking	Individual uses its tongue to lick an area close to a scent-gland on its own body.

**Table 2:** Locations and definitions of possible scent-mark deposition.

Deposition Location	Description
Branches	Natural structures in the enclosure consisting of logs, trees and various plant species.
Hatches	Small entrances that can be open or closed, linking the different enclosures together.
Climbing Frames	Man-made wooden structures in enclosure consisting of walkways, ropes and platforms.
Enrichment Devices	Removable objects that were placed daily to promote good welfare.
Ground	The artificial floor of the indoor enclosures and the grassy area of the outdoor enclosure.

**Table 3:** Volatile compounds present in filter paper samples from male and female ano-genital odour secretions identified tentatively using ChemStation (Agilent Technologies) and NIST (version MSD F.01.01.2317) mass spectral databases, listed in order of retention time. Compounds in **bold font** were found in both male and female samples.

Molecular Weight (Da)	Compound	Sex
94.042	Phenol	Female
**100.089**	**Hexanal**	**Both**
**106.042**	**Benzaldehyde**	**Both**
106.078	Benzene, 1,3-dimethyl-	Male
107.063	4-Cyanocyclohexene	Female
114.104	Heptanal	Male
118.099	Ethanol, 2-butoxy-	Female
108.058	Benzyl alcohol	Female
122.037	Phenol, 4-ethyl-	Female
126.104	5-Hepten-2-one, 6-methyl-	Male
126.104	2-Octenal, (E)-	Male
**128.120**	**1-Octen-3-ol**	**Both**
128.120	3-Octanone	Female
**130.000**	**2-Ethyl- 1-Hexanol**	**Both**
136.125	.beta.-Myrcene	Female
**136.125**	**D-Limonene**	**Both**
136.125	.beta.-Ocimene	Female
136.158	.alpha.-Pinene	Female
138.104	Furan, 2-pentyl-	Male
**142.136**	**Nonanal**	**Both**
152.047	Methyl salicylate	Female
154.136	Linalool	Female
154.136	Terpinen-4-ol	Female
154.136	α-Terpineol	Female
156.151	7-Octen-2-ol, 2,6-dimethyl-	Female
**156.151**	**Decanal**	**Both**
158.167	1-Decanol	Female
182.203	Cyclohexane, 2-butyl-1,1,3-trimethyl-	Female
184.119	Undecane, 2,6-dimethyl-	Male
184.219	Dodecane, 4-methyl-	Male
196.143	Linalyl acetate	Male
196.146	4-Hexen-1-ol, 5-methyl-2-(1-methylethenyl)-, acetate	Female
198.235	Tridecane, 6-methyl-	Female
214.099	Benzene, 1,1′-[1,2-ethanediylbis(oxy)]bis-	Male
248.114	2-Bromo dodecane	Female
**286.214**	**2,2,4-Trimethyl-1,3-pentanediol diisobutyrate**	Male
356.329	Carbonic acid, decyl undecyl ester	**Both**
426.407	Carbonic acid, decyl hexadecyl ester	Male
*-*	*Unknown hydrocarbon 01*	Male
*-*	*Unknown hydrocarbon 02*	Male
*-*	*Unknown hydrocarbon 03*	Male
*-*	*Unknown hydrocarbon 04*	Male
*-*	*Unknown hydrocarbon 05*	Male
*-*	*Unknown hydrocarbon 06*	Male
*-*	*Unknown hydrocarbon 07*	Male
*-*	*Unknown hydrocarbon 08*	Male
*-*	*Unknown hydrocarbon 09*	Male
*-*	*Unknown hydrocarbon 10*	Male
-	*Unknown hydrocarbon 11*	Male
-	*Unknown hydrocarbon 12*	Male
-	*Unknown hydrocarbon 13*	Male
-	*Unknown hydrocarbon 14*	Male

## Data Availability

The data presented in this study are available on request from the corresponding author.
